# *Tetracera loureiri* Extract Regulates Lipopolysaccharide-Induced Inflammatory Response Via Nuclear Factor-κB and Mitogen Activated Protein Kinase Signaling Pathways

**DOI:** 10.3390/plants11030284

**Published:** 2022-01-21

**Authors:** Jung A Lee, Ju Young Shin, Seong Su Hong, Young-Rak Cho, Ju-Hyoung Park, Dong-Wan Seo, Joa Sub Oh, Jae-Shin Kang, Jae Ho Lee, Eun-Kyung Ahn

**Affiliations:** 1 Bio-Center, Gyeonggido Business and Science Accelerator (GBSA), Suwon 16229, Korea; lovelee90@gbsa.or.kr (J.A.L.); bestgene@gbsa.or.kr (S.S.H.); yrcho@gbsa.or.kr (Y.-R.C.); 2 College of Pharmacy, Dankook University, Cheonan 31116, Korea; jjoosjy@gmail.com (J.Y.S.); jhp0607@dankook.ac.kr (J.-H.P.); dwseomb@dankook.ac.kr (D.-W.S.); jsoh@dankook.ac.kr (J.S.O.); 3 Biological Genetic Resources Utilization Division, National Institute of Biological Resources, Incheon 22689, Korea; diatom@korea.kr (J.-S.K.); leejaeho@korea.kr (J.H.L.)

**Keywords:** *Tetracera loureiri*, inflammation, RAW264.7 macrophages, NF-κB, MAPK

## Abstract

*Tetracera loureiri* (*T. loureiri*) is a woody climber inhabiting open deciduous or evergreen forests in Southeast Asia. A decoction comprising its stem and other herbs is a traditional Thai remedy for fatigue and jaundice, as well as to promote overall health. Anti-inflammatory effects induced by *T. loureiri* extract have not been reported. In this study, we investigated the anti-inflammatory effect of an ethanol extract of *T. loureiri* (ETL) on lipopolysaccharide (LPS)-induced inflammatory response in RAW264.7 macrophages. We found that ETL treatment inhibited the production of nitric oxide (NO) in LPS-stimulated RAW264.7 cells, without affecting cell viability. The effect of ETL on the expression of various pro-inflammatory mediators was analyzed using reverse transcription-polymerase chain reaction (RT-PCR), Western blotting, and enzyme-linked immunosorbent assay (ELISA). We observed that ETL inhibited the expression of inducible nitric oxide synthase (iNOS) and cyclooxygenase-2 (COX-2) at the mRNA and protein levels and decreased the production of prostaglandin E_2_ (PGE_2_) by COX-2 in RAW264.7 macrophages. ETL dose-dependently reduced the production of pro-inflammatory cytokines including tumor necrosis factor-α (TNF-α), interleukin-1β (IL-1β), and interleukin-6 (IL-6) in LPS-induced RAW264.7 cells, in a dose-dependent manner. Furthermore, ETL suppressed the LPS-induced nuclear translocation of the nuclear factor, NF-κB. Additionally, ETL was found to inhibit the activation of mitogen-activated protein kinases (MAPK), such as extracellular signal-regulated kinase, c-Jun-N-terminal kinase, and p38 MAPK. In conclusion, our findings demonstrate that ETL inhibits the expression of pro-inflammatory mediators and cytokines, thereby downregulating NF-κB and MAPK signaling pathways in LPS-stimulated macrophages, Consequently, ETL is a potential therapeutic agent for the treatment of inflammatory diseases.

## 1. Introduction

*Tetracera*, a genus in the Dilleniaceae family, comprises about 50 species, including *Tetracera loureiri. T. loureiri* is a herb used in traditional medicine in Southeast Asia, including Cambodia and Thailand. In traditional folk medicine, it has been used as a diuretic agent and in the treatment of jaundice [1]. It has been reported to possess antioxidant and free radical scavenging properties. Previous phytochemical investigations of this plant revealed the presence of acylated-triterpenoid, flavonoids, and lignans in the stems [2]. These compounds exhibited multiple biological activities, such as anti-cancer, anti-HIV, and bacterial biofilm inhibition [3,4,5]. As demonstrated in pharmacological studies, it prevents elevation of plasma ALT and AST levels in vivo, through its hepatoprotective effect [6]. Along with antioxidant activities, high polarity extracts also exhibit α-amylase, and α-glucosidase inhibitory activities [7]. However, the anti-inflammatory property of *T. loureiri* extract has not been studied so far.

Inflammation is a defense mechanism against infection or pathogen entry into the body. It is a biological response involving the modulation of immune cells along with a variety of molecular mediators [8,9]. Macrophages enact complex immune responses, including immune monitoring, chemotaxis, and removal of target antigens. They are also involved in embryogenesis, wound healing, suicide elimination, and tissue remodeling during hematopoietic cell proliferation. Upon activation, macrophages induce an inflammatory response, which stimulates nitric oxide (NO) and prostaglandin E_2_ (PGE_2_) production and increases cytokine levels, including that of tumor necrosis factor alpha (TNF-α), interleukin-1β (IL-1β), and IL-6 [10,11,12]. Nuclear factor-κB (NF-κB), an important transcription factor consisting of p50 and p65 subunits, modulates the synthesis of a variety of cytokines, chemokines, and growth factors. Once NF-κB is phosphorylated and translocated to the nucleus, it leads to the synthesis of inducible nitric oxide synthase (iNOS), cyclooxygenase-2 (COX-2), and various pro-inflammatory cytokines. The expression of pro-inflammatory cytokines and NF-κB is further controlled by mitogen-activated protein kinases (MAPKs), such as extracellular signal-regulated kinase (ERK), p38 kinase (p38), and c-Jun N-terminal kinase (JNK) [13,14,15,16].

In this study, we investigated the anti-inflammatory effect of quercetin and rhamnocitrin isolated from an ethanol extract of *T. loureiri* (ETL) on LPS-stimulated inflammatory responses in RAW264.7 cells. Furthermore, we demonstrated the reduction in NO production and the inhibition of the expression of iNOS, COX-2, PGE_2_, and pro-inflammatory cytokines caused by ETL, through the suppression of the NF-κB and MAPK signaling pathways.

## 2. Results

### 2.1. Effect of ETL on Cell Viability and NO Production in LPS-Stimulated RAW264.7 Cells

**Figure 1:**
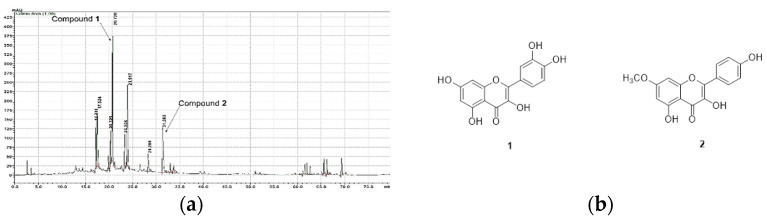
High Pressure Liquid Chromatography (HPLC) of an ethanol extract of *T. loureiri* (ETL) and chemical structures of two ETL compounds. (**a**) HPLC chromatogram (at 330 nm) of ETL. (**b**) Structures of quercetin (**1**) and rhamnocitrin (**2**).

**Figure 2:**
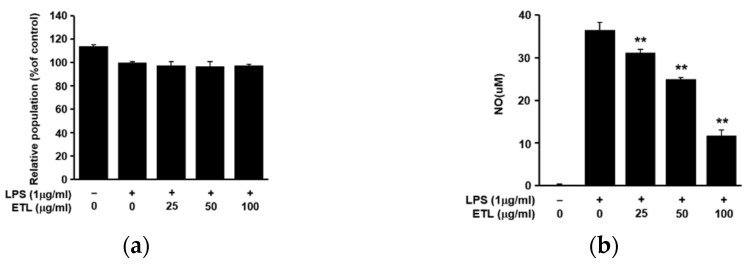
Effect of ETL on cell viability and NO production in LPS-stimulated RAW264.7 cells. The cells were pre-treated with ETL (25–100 μg/mL) for 1 h, followed by LPS (1 μg/mL) treatment for 24 h (the untreated control/LPS(-), the LPS-treated group/LPS(+)). (**a**) Cell viability, as determined using the MTT assay. (**b**) NO production in the cell culture supernatant, as measured using Griess reagent. Data are expressed as the mean ± standard deviation (SD) of three replicates; ** *p* < 0.01, compared to the LPS-treated cells.

*T. loureiri* stems were pulverized and dried, and the target compounds were extracted using 70% aqueous ethanol and analyzed using high-performance liquid chromatography (HPLC) and medium pressure liquid chromatography (MPLC) (Figure 1a). *T. loureiri* contains two compounds, quercetin and rhamnocitrin. The structures of quercetin and rhamnocitrin are shown in Figure 1b. Although quercetin has been widely known for a long time, it was isolated and identified from *T. loureiri* for the first time [2]. We determined the cytotoxic effect of ETL in the LPS-induced inflammatory response, using the MTT assay. ETL treatment did not show any significant cytotoxic effect on LPS-stimulated RAW264.7 cells (Figure 2a). We determined the inhibitory effect of ETL on NO production in LPS-stimulated RAW264.7 cells. As demonstrated in Figure 2b, ETL treatment significantly inhibited NO production in LPS-stimulated RAW264.7 cells in a dose-dependent manner. Treatment with 50 and 100 μg/mL of ETL decreased the NO concentration by 31.7% and 67.9%, respectively. These results indicate that ETL suppresses NO production without inducing cytotoxicity in LPS-stimulated macrophages.

### 2.2. Effect of ETL on the Expression of iNOS, COX-2 and PGE_2_ Production

**Figure 3:**
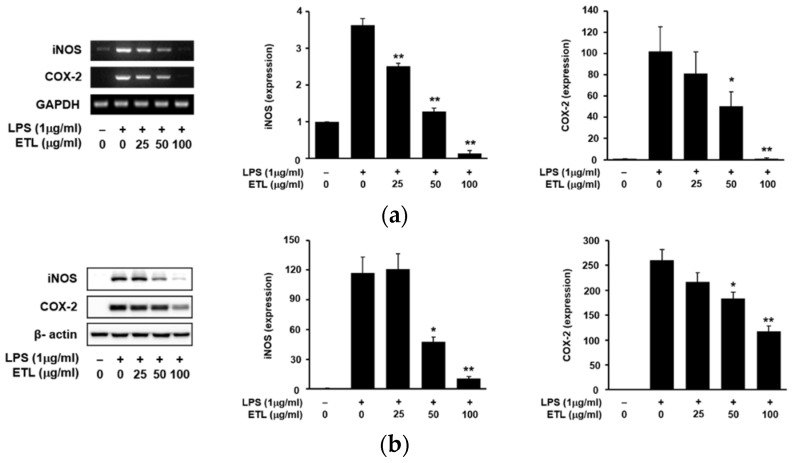
Effect of ETL on the mRNA and protein expression of iNOS and COX-2 in LPS-stimulated RAW264.7 cells. The cells were pre-treated with ETL (25–100 μg/mL) for 1 h, followed by LPS (1 μg/mL) treatment for 24 h (the untreated control/LPS(-), the LPS-treated group/LPS(+)). (**a**) mRNA expression of iNOS and COX-2, as evaluated by RT-PCR analysis. Glyceraldehyde 3-phosphate dehydrogenase (GAPDH) was used as the internal control. (**b**) Protein expression of iNOS and COX-2, as determined by Western blot analysis. β-actin was used as the internal control. Data are expressed as the mean ± SD of three replicates; * *p* < 0.05 and ** *p* < 0.01, compared to the LPS-treated cells.

**Figure 4:**
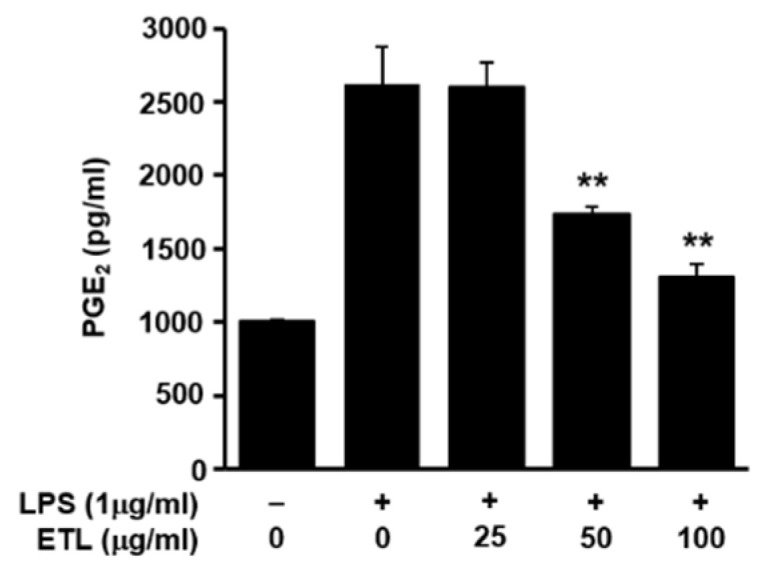
Effect of ETL on the production of PGE_2_ in LPS-stimulated RAW264.7 cells. The cells were pre-treated with ETL (25–100 μg/mL) for 1 h, followed by LPS (1 μg/mL) stimulation for 24 h (the untreated control/LPS(-), the LPS-treated group/LPS(+)). Analysis of PGE_2_ production in the cell culture supernatant, as determined using enzyme-linked immunosorbent assay (ELISA). Data are expressed as the mean ± SD of three replicates; ** *p* < 0.01, compared to the LPS-treated cells.

An increase in the expression of iNOS induces the production of NO in LPS-stimulated cells. COX-2 stimulates the production of PGE_2_. Therefore, the anti-inflammatory effect of ETL was evaluated by inhibiting the expression of iNOS and COX-2 [17,18]. We examined the effects of ETL on iNOS and COX-2 expression in LPS-stimulated cells by reverse transcription-polymerase chain reaction (RT-PCR) and Western blot analysis. Both iNOS and COX-2 play an important role in LPS-induced NO production, in the inflammatory response [19]. As shown in Figure 3, ETL treatment inhibited the expression of LPS-induced iNOS and COX-2 at the mRNA level in RAW264.7 cells, in a dose-dependent manner. Additionally, we found that ETL reduced the protein levels of both iNOS and COX-2. We also confirmed the effect of ETL on the production of PGE_2_, which is an inflammatory mediator produced by COX-2 in RAW264.7 macrophages [13]. The production of PGE_2_ when cells were treated with ETL decreased in a dose-dependent manner in RAW264.7 cells, compared to that in LPS-treated cells. ETL inhibited the production of PGE_2_ by 33.6% and 50.1% (Figure 4) in cells treated with 50 μg/mL and 100 μg/mL of ETL, respectively.

### 2.3. Effect of ETL on the Production of Pro-Inflammatory Cytokines in LPS-Stimulated RAW264.7 Cells

**Figure 5:**
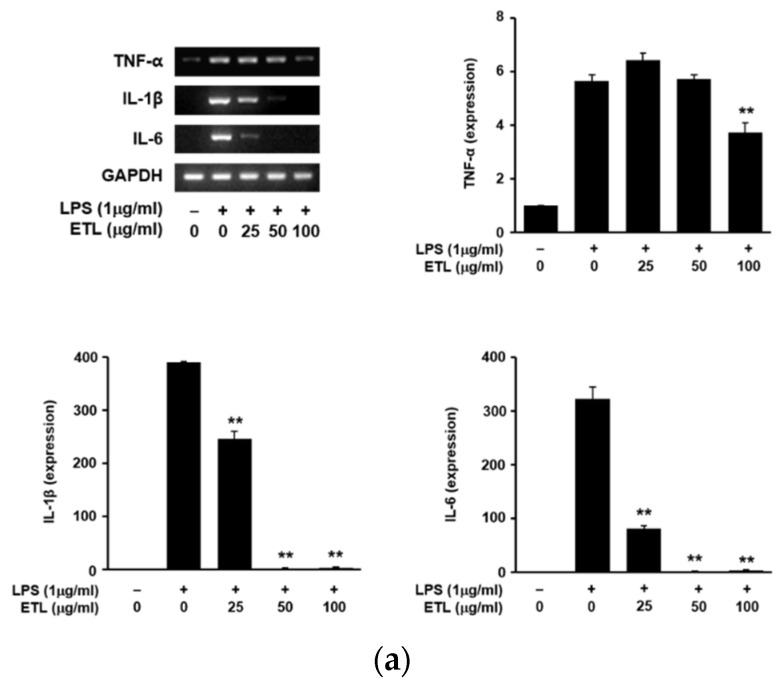

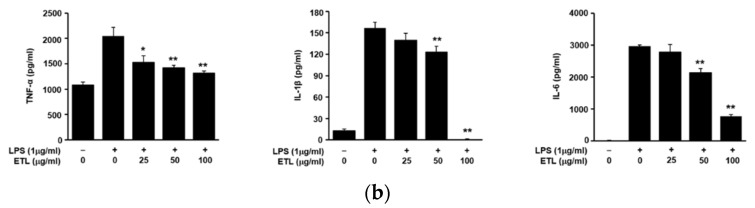
Effect of ETL on the expression of TNF-α, IL-1β, and IL-6 in LPS-stimulated RAW264.7 cells. Cells were pre-treated with ETL (25–100 μg/mL) for 1 h, followed by LPS (1 μg/mL) treatment for 24 h (the untreated control/LPS(-), the LPS-treated group/LPS(+)). (**a**) mRNA expression of TNF-α, IL-1β, and IL-6, as analyzed by RT-PCR. (**b**) Pro-inflammatory cytokine levels in the cell culture supernatant, determined using ELISA. Data are expressed as the mean ± SD of three replicates; * p < 0.05 and ** *p* < 0.01, compared to the LPS-treated cells.

Cytokines like TNF-α, IL-1β, and IL-6 are considered pro-inflammatory cytokines as they are involved in the regulation of inflammatory responses. This study investigated any inhibitory effects of ETL on these pro-inflammatory mediators. We examined the release of TNF-α, IL-1β, and IL-6 in LPS-induced cells, using RT-PCR and ELISA [20]. We found that ETL treatment significantly decreased the expression of TNF-α, IL-1β, and IL-6 at the mRNA level (Figure 5a). Furthermore, ETL reduced the level of these cytokines in cell supernatants, in a dose-dependent manner (Figure 5b). Treatment with an effective dose of 100 μg/mL of ETL inhibited the production of TNF-α, IL-1β, and IL-6 by 35.5%, 99.6%, and 74.0%, respectively. These results suggest that ETL effectively controls the induction of inflammation and related factors by inhibiting the production of pro-inflammatory cytokines.

### 2.4. Effect of ETL on the Nuclear Translocation of NF-κB in LPS-Stimulated RAW264.7 Cells

**Figure 6:**
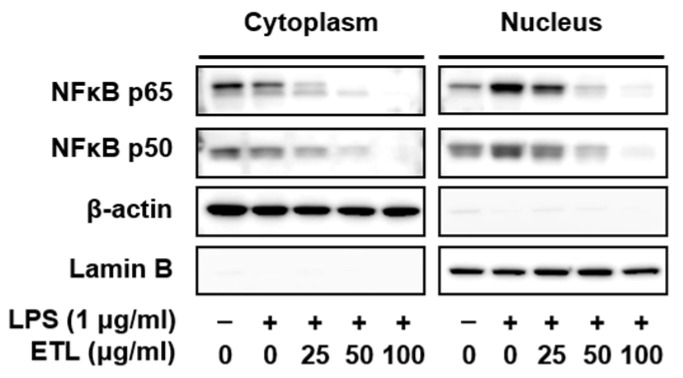
Effect of ETL on NF-κB nuclear localization in LPS-stimulated RAW264.7 cells. Cells were pre-treated with ETL (25–100 μg/mL) for 1 h and then treated with LPS (1 μg/mL) for 30 min (the untreated control/LPS(-), the LPS-treated group/LPS(+)). The cytosolic and nuclear extracts were subjected to Western blot analysis using anti-NF-κB p65, anti-NF-κB p50, anti-β-actin (cytosolic fraction loading control), and anti-lamin B (nuclear fraction loading control) antibodies. Data are expressed as the mean ± SD of three replicates.

NF-κB is a transcription factor involved in various pathways like cytokine response, inflammation, and cell growth regulation. It has been reported to be involved in promoting the production of major pro-inflammatory factors like TNF-α, IL-1β, and IL-6 [21,22]. Upon activation, NF-κB activates the genes of various inflammation-associated targets like iNOS, COX-2, TNF-α, IL-1β, and IL-6 in LPS-stimulated cells [23]. To verify the involvement of ETL in the NF-κB pathway, we investigated the nuclear translocation of NF-κB (p65 and p50) from the cytosol, in LPS-stimulated RAW264.7 macrophages, using Western blot analysis. As shown in Figure 6, ETL markedly reduced the LPS-induced nuclear translocation of NF-κB (p65 and p50). In this experiment, β-actin was used as the cytoplasmic control and lamin B was used as the nuclear control. Our findings suggest that ETL inhibits the NF-κB signaling pathway by blocking the LPS-induced nuclear translocation of the p65 and p50 subunits.

### 2.5. Effect of ETL on LPS-Stimulated MAPK Phosphorylation

**Figure 7:**
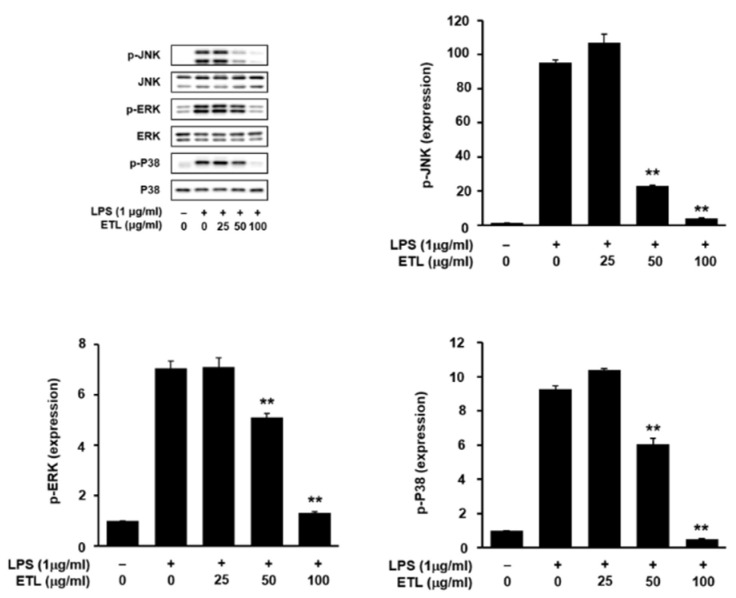
Effect of ETL on the phosphorylation of JNK, ERK, and p38 in LPS-stimulated RAW264.7 cells. The cells were pre-treated with ETL (25–100 μg/mL) for 1 h and then treated with LPS (1 μg/mL) for 30 min (the untreated control/LPS(-), the LPS-treated group/LPS(+)). The cell lysates were subjected to Western blot analysis using anti-phospho-JNK, anti-JNK, anti-phospho-ERK, anti-ERK, anti-phospho-p38, and anti-p38 antibodies. The results shown are representative of at least three independent experiments; ** *p* < 0.01, compared to the LPS-treated cells.

MAPKs are the one of the most important signaling factors involved in pathways associated with cell growth, differentiation, and regulation of cellular response. Additionally, they are involved in the production of various inflammatory mediators, and they affect the activation of NF-κB [24,25,26]. Here, using Western blot analysis, we determined the inhibitory effect of ETL on the phosphorylation of JNK, ERK, and p38 MAPK, which are involved in the LPS-induced inflammatory response in RAW264.7 macrophages. As presented in Figure 7, ETL suppressed the phosphorylation of JNK, ERK, and p38 MAPK in LPS-stimulated RAW264.7 cells, in a dose-dependent manner. These findings suggest that the inhibitory effect of ETL on the LPS-stimulated inflammatory response may be due to the inhibition of MAPK phosphorylation in RAW264.7 cells.

### 2.6. Anti-Inflammatory Effects of Quercetin and Rhamnocitrin Isolated from ETL on RAW264.7 Cells

**Figure 8:**
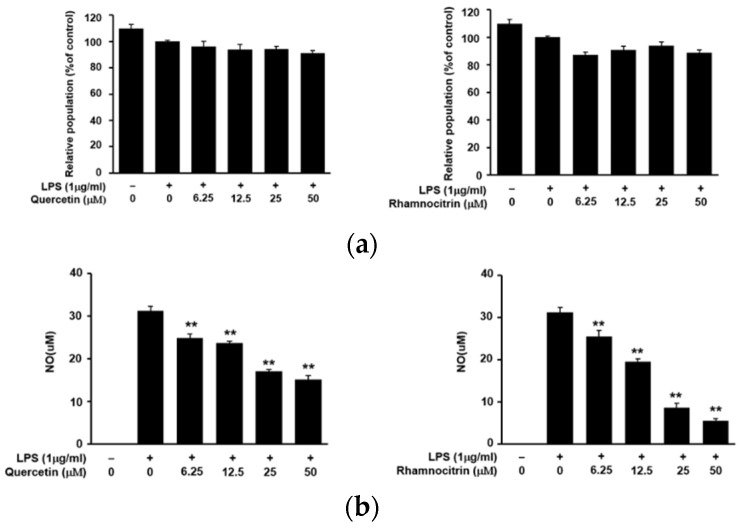
Effect of quercetin and rhamnocitrin on cell viability and NO production in LPS-stimulated RAW264.7 cells. The cells were pre-treated with quercetin and rhamnocitrin (6.25–50 μM total) for for 1 h, followed by LPS (1 μg/mL) treatment for 24 h (the untreated control/LPS(-), the LPS-treated group/LPS(+)). (**a**) Cell viability, as determined by MTT assay. (**b**) NO production in the cell culture supernatant, as measured using Griess reagent. Data are expressed as the mean ± SD of three replicates; ** *p* < 0.01, compared to the LPS-treated cells.

To confirm the potential anti-inflammatory activity of ETL, we purified and identified the functional compounds in the *T. loureiri* extract. A total of 12 compounds were identified: betulinic acid, *erythro*-carolignan E, *threo*-carolignan E, eucalyptolic acid, 3β-*O*-*trans*-feruloyl-2α-hydroxyurs-12-en-28-oic acid, 3β-*O*-(*trans*-*ρ*-coumaroyl) maslinic acid, jacoumaric acid, quercetin, europetin, kaempferol, rhamnetin, and rhamnocitrin [2]. Among these compounds, we confirmed that quercetin and rhamnocitrin inhibited NO production upon LPS-stimulation in RAW264.7 cells, in a dose-dependent manner. Treatment with 50 μM each of quercetin and rhamnocitrin decreased the NO concentration by 48.6 and 17.8%, respectively. Furthermore, neither of the two compounds (6.25, 12.5, 25, 50 µM total) exhibited any cytotoxic effect (Figure 8). These results indicate that quercetin and rhamnocitrin are important compounds for the anti-inflammatory nature of ETL.

## 3. Discussion

Various biochemical mechanisms and factors are involved in the generation of an inflammatory response. Among them, macrophages, one of the key factors, act through the production of NO, along with various pro-inflammatory mediators, such as iNOS, COX-2, PGE_2_, and pro-inflammatory cytokines [27,28].

Previous studies on *T. loureiri* have reported on its antioxidant activity, hepato-protective effect, and free radical scavenging property [6]. In the present study, we assessed the anti-inflammatory effect of *T. loureiri* on macrophages. First, we demonstrated the dose-dependent inhibition of NO production by ETL in RAW264.7 macrophages, without the induction of cytotoxicity (Figure 2). The active compounds isolated from this extract were identified as quercetin and rhamnocitrin; these compounds are being reported in *T. loureiri* for the first time. On examination, we found that these two compounds also suppressed NO production, without inducing cytotoxicity, in LPS-stimulated macrophages (Figure 8).

In the current study, we demonstrated the inhibitory effect of ETL on the expression of iNOS and COX-2 at the mRNA and protein levels (Figure 3), in LPS-stimulated macrophages, and showed a reduction in the level of PGE_2_ that is synthesized by COX-2 (Figure 4). iNOS and COX-2 are the major macrophage-derived inflammatory mediators [28,29]. NO is produced by three isoforms of NOS. The level of iNOS, an isoform of NOS, is increased by LPS-stimulation or by pro-inflammatory cytokines. COX-2 is involved in the synthesis of PGE_2_ [29,30]. We observed that ETL decreased the levels of various pro-inflammatory cytokines, including TNF-α, IL-1β, and IL-6, in LPS-treated RAW264.7 cells (Figure 5). TNF-α, IL-1β, and IL-6 are the most important pro-inflammatory cytokines in an inflammatory response. TNF-α is produced mainly by activated macrophages, and its production is increased by LPS-stimulation. It is also produced in various cells, like mast cells, lymphoid cells, NK cells, eosinophils, and endothelial cells [31,32]. IL-1β and IL-6 are factors necessary for cell growth and homeostasis when present in low concentrations. Conversely, during an inflammatory response, they are secreted in large quantities, resulting in aggravated symptoms. The primary role of TNF-α, IL-1β, and IL-6 is the regulation of inflammatory response when wounded, and during infection or immune stimulation [6,33]. Therefore, the inhibition of pro-inflammatory mediators including cytokines is essential for the control of an inflammatory response.

This finding led us to investigate the effect of ETL on the expression of transcription factors associated with inflammation. The transcription factor NF-κB plays an important role in regulating the gene expression of inflammatory factors [34]. NF-κB is a heterodimer composed of the p50 and p65 subunits. Following activation, NF-κB induces the expression of iNOS and COX-2 at the gene level and regulates their nuclear translocation [35]. According to many reports, LPS stimulation leads to NF-κB activation, which is associated with the MAPK signaling pathway. According to many reports, LPS stimulation leads to NF-κB activation, which is associated with the MAPK signaling pathway. MAPKs are members of important signaling pathways in the inflammatory response. As a result of the inflammatory response, there is increased phosphorylation of ERK, JNK, and p38 in LPS-stimulated RAW264.7 cells. The activation of MAPKs, including ERK, JNK, and p38, is associated with pro-inflammatory cytokines [15,24,25,26]. In our study, we found that ETL treatment suppressed NF-κB activation and the translocation of its p65 and p50 subunits. Additionally, it inhibited the phosphorylation of JNK, ERK, and p38 (Figure 6 and Figure 7). These findings suggest that ETL imposes an anti-inflammatory response by inhibiting the NF-κB and MAPK signaling pathways.

In conclusion, the present study suggested that ETL opposes inflammation via the inhibition of the expression of iNOS, COX-2, and cytokines, along with the downregulation of the NF-κB and MAPK signaling pathways, in LPS-stimulated macrophages. In addition, this study is the first to show that quercetin and rhamnocitrin, isolated from ETL, possess anti-inflammatory activity. These results suggest that ETL and its compounds are potential candidates for the development of anti-inflammatory drugs to help prevent inflammatory diseases.

## 4. Materials and Methods

### 4.1. Plant Materials

The dried stems of *T. loureiri* were obtained from Thnong, Kandol, Botum Sarkor, Koh kong, Cambodia, in December 2014, and were identified by Dr. Jae-Shin Kang (Biological Genetic Resources Utilization Division, National Institute of Biological Resources, Incheon, Republic of Korea). A voucher specimen (#153) of this plant was deposited at the Bio-Center, Gyeonggido Business and Science Accelerator (GBSA), Suwon, Republic of Korea.

### 4.2. Preparation of T. loureiri Extract

*T. loureiri* stems (1 kg) were pulverized, and the dry material was percolated with 70% aqueous ethanol for 24 h at 20–22 °C. The extract was filtered and concentrated under vacuum and reduced pressure (temperature, 40 °C; pressure, 10 hPa) using a rotary flash evaporator (Büchi Labortechnik AG, Flawil, Switzerland), allowing for the complete evaporation of ethanol. The remaining aqueous solution was concentrated under vacuum (temperature, −85 °C; pressure, 5 mTorr) and freeze-dried. The yield of the crude *T. loureiri* stems extract was 3.88% (*w*/*w*). After solvent evaporation under reduced pressure, the residues were suspended in water and then successively partitioned using n-hexane, CH_2_Cl_2_, EtOAc, and *n*-BuOH to acquire yields of 14.6, 23.4, 47.1, and 91.0 g, respectively. The EtOAc-soluble layer was subjected to reverse-phase silica gel flash column chromatography (MeOH/water gradient, 3:7 to 1:0) to acquire six sub-fractions (#153E-1~6). Fraction #153E-4 (2.94 g) was subjected to HPLC [MeOH/water (0.05% trifluoroacetic acid) gradient, 45:55 to 9:1; 30 min] to yield compound **1** (quercetin, 35.4 mg). Compound **2** (rhamnocitrin, 10.8 mg) was isolated from fraction #153E-6 (1.44 g) using MPLC [column: silica gel, 40 g; 40 mL/min; CH_2_Cl_2_/MeOH = 1:0 to 1:1; 45 min].

Quercetin (compound **1**): yellow amorphous powder; ^1^H-NMR (700 MHz, DMSO-d_6_) δ 12.49 (1H, s, 5-OH), 10.77 (1H, s, 7-OH), 9.58 (1H, s, 4′-OH), 9.36 (1H, s, 3-OH), 9.30 (1H, s, 3′-OH), 7.67 (1H, d, *J* = 2.1, H-2′), 7.54 (1H, dd, *J* = 8.4, 2.1, H-6′), 6.88 (1H, d, *J* = 8.4, H-5′), 6.40 (1H, d, *J* = 2.1, H-8), 6.18 (1H, d, *J* = 2.1, H-6); ^13^C-NMR (175 MHz, DMSO-d_6_) δ 175.8 (C-4), 163.9 (C-7), 160.7 (C-5), 156.1 (C-9), 147.7 (C-4′), 146.8 (C-2), 145.0 (C-3′), 135.7 (C-3), 121.9 (C-1′), 119.9 (C-6′), 115.6 (C-5′), 115.0 (C-2′), 103.0 (C-10), 98.2 (C-6), 93.3 (C-8); ESI-MS (*m*/*z*) 303 [M + H]^+^ (Figures S1–S3). The structure of quercetin is presented in Figure 1b [2].

Rhamnocitrin (compound **2**): yellow amorphous powder; ^1^H-NMR (700 MHz, DMSO-d_6_) δ 12.49 (1H, s, 5-OH), 10.15 (1H, s, 4′-OH), 9.53 (1H, s, 3-OH), 8.10 (2H, d, *J* = 8.4, H-2′, 6′), 6.94 (2H, d, *J* = 8.4, H-3′, 5′), 6.76 (1H, d, *J* = 2.1, H-8), 6.36 (1H, d, *J* = 2.1, H-6); ^13^C-NMR (175 MHz, DMSO-d_6_) δ 176.0 (C-4), 164.9 (C-7), 160.4 (C-5), 159.3 (C-4′), 156.1 (C-9), 147.2 (C-2), 136.0 (C-3), 129.6 (C-2′, 6′), 121.6 (C-1′), 115.5 (C-3′, 5′), 104.0 (C-10), 97.5 (C-6), 92.0 (C-8), 56.0 (7-OCH_3_); ESI-MS (*m*/*z*) 299 [M − H]^−^ (Figure S4–S6). The structure of rhamnocitrin is presented in Figure 1b [2].

### 4.3. Apparatus and Chromatography Conditions

HPLC analysis was performed on a SHIMADZU (Shimadzu Scientific Instrument Incorporated, Kyoto, Japan) system consisting of an LC-20AT pump, a CTO-20A thermostat column compartment, and SPD-M20A diode array detector. Separation was performed on a Kromacil C18 column (250 × 4.6 mm internal diameter, 5 μm particle size) (SHISEIDO Co., Tokyo, Japan). The mobile phase consisted of water–TFA (99.95:0.05; *v*/*v*) (solvent A) and acetonitrile (solvent B). Elution was performed using the following gradient: initial—90:10 (A:B; *v*/*v*); post 40 min—60:40 (A:B; *v*/*v*); post 60 min—0:100 (A:B; *v*/*v*). The mobile phase was freshly prepared, filtered through a 0.45 mm, WTP 0.5 mm membrane (Whatman, Maidstone, UK), sonicated prior to use, and delivered at a flow rate of 1.0 mL/min. The injection volume was 10 μL, and the column temperature was 35 °C. All operations, including the acquisition and analysis of data, were controlled by LabSolutions, LC system software (Shimadzu Scientific Instrument Incorporated, Kyoto, Japan).

### 4.4. Cell Culture

Murine macrophage RAW264.7 cells (TIB-71) were obtained from the American Type Culture Collection (ATCC, Manassas, VA, USA). The cells were cultured at 37 °C with 5% CO_2_ in Dulbecco’s modified Eagle’s medium (DMEM) containing 10% fetal bovine serum, 100 U/mL of penicillin, and 0.1 mg/mL of streptomycin (Thermo Fisher Scientific, Inc., Waltham, MA, USA).

### 4.5. Cell Viability Assay

The viability of murine macrophage RAW264.7 cells was determined using the 3-[4,5-dimethylthiazol-2-yl]-2,5-diphenyltetrazolium bromide (MTT; Duchefa Biochemie B. V., Haarlem, Netherlands) assay. RAW264.7 cells were seeded at a density of 5 × 10^4^ cells/well in a 96-well plate. Following incubation for 24 h, the cells were treated with ETL (25, 50, 100 μg/mL) or quercetin and rhamnocitrin (6.25, 12.5, 25, 50 μM) for 1 h and treated with 1 μg/mL LPS (Sigma Aldrich, St. Louis, MO, USA) or left untreated for 24 h. The medium was removed, and MTT solution (5 mg/mL in PBS) was added to each well, followed by incubation for 2 h. The supernatant was removed and dimethyl sulfoxide (DMSO; Duchefa Biochemie B. V.) was added to each well. Subsequently, the plate was shaken to dissolve the formazan formed. Absorbance was measured at 540 nm using a SpectraMax 190PC microplate reader (Molecular Devices, Sunnyvale, CA, USA) [36,37].

### 4.6. Measurement of NO Production

RAW264.7 cells were seeded at a density of 5 × 10^4^ cells/well in a 96-well plate. Following incubation for 24 h, the cells were treated with ETL (25, 50, 100 μg/mL) or quercetin and rhamnocitrin (6.25, 12.5, 25, 50 μM) for 1 h and treated with 1 μg/mL LPS or remained untreated for 24 h (untreated control cells without LPS remained as the control group) [38,39]. The amount of NO generated was analyzed using the Griess reaction. Equal volumes of cultured medium and Griess reagent (Sigma Aldrich, St. Louis, MO, USA) were mixed and used to incubate the cells in at room temperature for 10 min. Subsequently, absorbance was measured at 540 nm using a SpectraMax 190PC microplate reader.

### 4.7. ELISA

RAW264.7 cells were seeded at a density of 1 × 10^6^ cells/well in a 6-well plate. Following incubation for 24 h, the cells were treated with ETL (25, 50, 100 μg/mL) for 1 h and treated with 1 μg/mL LPS or left untreated for 24 h. The concentrations of PGE_2_ (cat. no. KGE004B, R&D Systems, Minneapolis, MN, USA) and the pro-inflammatory cytokines, TNF-α (cat no. BMS607/3, Invitrogen, Thermo Fisher Scientific, Inc., Waltham, MA, USA), IL-1β (cat. no. BMS6002, Invitrogen), and IL-6 (cat. no. BMS614/2, Invitrogen) in the culture medium were measured using ELISA, according to the manufacturer’s instructions.

### 4.8. Reverse Transcription-Polymerase Chain Reaction (RT-PCR)

RAW264.7 cells were seeded at a density of 1 × 10^6^ cells/well in a 6-well plate. Following incubation for 24 h, the cells were treated with ETL (25, 50, 100 μg/mL) for 1 h and treated with 1 μg/mL LPS or left untreated for 24 h. The cells were harvested and washed using PBS, and the total RNA was extracted using TRIzol reagent (Invitrogen). cDNA synthesis was performed on 1 μg of the extracted total RNA using the SuperScript^®^III first-strand synthesis system (Invitrogen). The cDNA was amplified using specific primers and AccuPower^®^ Pfu PCR premix (Bioneer Corporation, Daejeon, Republic of Korea). The following conditions were used for the PCR reaction: 95 °C for 5 min (1 cycle); 95 °C for 30 s, 55 °C for 40 s, and 72 °C for 1 min (30 cycles); and final extension at 72 °C for 10 min. The primer (Bioneer Corporation, Daejeon, Republic of Korea) sequences used for RT-PCR are shown in Table 1. The band intensity was visualized on a ChemiDoc XRS system using the Quantity One software version 4.6.3 (Bio-Rad Laboratories, Inc., Hercules, CA, USA). GAPDH was used as the invariant control.

### 4.9. Preparation of Nuclear Extract

RAW264.7 cells were washed using PBS and harvested. The cells were re-suspended in 200 μL lysis buffer (10 mM HEPES at pH 7.9; 10 mM KCl; 1 mM DTT; 0.5 mM PMSF; and 0.1 mM EDTA) and centrifuged at 20,000× *g* for 5 min at 4 °C. Subsequently, 10% NP-40 was added and the cells were lysed on ice for 10 min. The cells were then centrifuged at 20,000× *g* for 2 min at 4 °C, and the supernatant was collected. This formed the cytosolic extract. The pellet was re-suspended in 50 μL extraction buffer (20 mM HEPES at pH 7.9; 0.4 M NaCl; 1 mM DTT; 1 mM PMSF; 1 mM EDTA; and 1% NP-40) and incubated on ice for 10 min. The nuclear extract was obtained by centrifugation at 15,000× *g* for 15 min at 4 °C.

### 4.10. Western Blot Analysis

RAW264.7 cells were seeded at a density of 1 × 10^6^ cells/well in a 6-well plate, treated with ETL (25, 50, 100 μg/mL) for 1 h, and treated with 1 μg/mL LPS for different time periods, as indicated in the legends of Figure 3 and Figure 7. The cells were lysed on ice for 30 min in RIPA buffer (Sigma Aldrich) consisting of a protease inhibitor and phosphatase inhibitors (Sigma Aldrich). The cells were centrifuged at 16,000× *g* for 30 min at 4 °C, and the supernatant was collected. The total proteins were separated on an 8% gel using sodium dodecyl sulfate polyacrylamide gel electrophoresis and transferred onto nitrocellulose membranes (Sigma Aldrich). Protein expression was analyzed by immunoblotting with antibodies against anti-iNOS (cat. no. ab3523; dilution, 1:500), anti-COX-2 (cat. no. ab3523, Abcam, Cambridge, UK) and anti-Lamin B (cat no. sc-6216, Santa Cruz Biotechnology, Inc., Dallas, TX, USA), β-actin (cat no. 5125), anti-NF-κB p65 (cat no. 8242), anti-NF-κB p50 (cat no. 12540), anti-phospho-JNK (T183/Y185; cat no. 4668), anti-JNK (cat no. 9252), anti-phospho-ERK (T202/Y204; cat no. 9101), anti-ERK (cat no. 9102), anti-phospho-p38 (T180/Y182; cat no. 9211), and anti-p38 (cat no. 9212) primary antibodies (dilution, 1:1000, Cell Signaling Technology, Inc. Danvers, MA, USA). Horseradish peroxidase-conjugated anti-rabbit antibodies (cat no. 7074; dilution, 1:2000; Cell Signaling Technology) and anti-goat antibodies (cat no. sc-2354; dilution, 1:5000, Santa Cruz Biotechnology) were used as secondary antibodies. The proteins were detected with SuperSignal^®^ West Pico chemiluminescent substrate (Thermo Fisher Scientific) using the Amersharm image 600 (GE Healthcare Life Sciences, Chicago, IL, USA).

### 4.11. Statistical Analysis

The data obtained were analyzed for statistical significance using a one-way analysis of variance (ANOVA) and Student’s *t*-test. The data are expressed as mean ± SD. * *p* < 0.05 and ** *p* < 0.01 were considered statistically significant.

## Figures and Tables

**Table 1:** Primer sequence for the reverse transcription-polymerase chain reaction.

Gene	Primer Sequences		Accession No.
TNF-α	forward reverse	5′-AGCCTGTAGCCCACGTCGTA-3′ 5′-TCTTTGAGATCCATGCCGTTG-3′	NM_013693
IL-1β	forward reverse	5′-CTTTGAAGAAGAGCCCATCC-3′ 5′-TTTGTCGTTGCTTGGTTCTC-3′	NM_008361
IL-6	forward reverse	5′-CACTTCACAAGTCGGAGGCTT-3′ 5′-GCAAGTGCATCATCGTTGTTC-3′	NM_031168
iNOS	forward reverse	5′-GAGTTCGAGACTTCTGTGA-3′ 5′-GGCGATCTGGTAGTAGTG-3′	NM_010927
COX-2	forward reverse	5′-GGAGAGACTATCAAGATAGTGATC-3′ 5′-ATGGTCAT AGACTTTTACAGCTC-3′	NM_011198
GAPDH	forward reverse	5′-GTATGACTCCACTCACGGCAAA-3′ 5′-GGTCTCGCTCCTGGAGAGATG-3′	NM_008084

## Data Availability

Not applicable.

## Supplementary Materials

The following are available online at https://www.mdpi.com/article/10.3390/plants11030284/s1, Figure S1: ^1^H NMR spectrum of **1**, Figure S2: ^13^C NMR spectrum of **1**, Figure S3: ESI-MS spectrum of **1**, Figure S4: ^1^H NMR spectrum of **2**, Figure S5: ^13^C NMR spectrum of **2**, Figure S6: ESI-MS spectrum of **2**.
