# Supplementary material for: Tetracera loureiri Extract Regulates Lipopolysaccharide-Induced Inflammatory Response Via Nuclear Factor-κB and Mitogen Activated Protein Kinase Signaling Pathways
Source: Plants (Basel). 2022 Jan 21;11(3):284. doi: 10.3390/plants11030284 (PMC8839383; doi:10.3390/plants11030284)
Supplement: Supplementary file 1 [file plants-11-00284-s001.zip › plants-1554076-supplementary.pdf]

Tetracera loureiri extract regulates lipopolysaccharide-induced inflammatory response via nuclear factor-κB and mitogen activated protein kinase signaling pathway

Jung A Lee[1, †], Ju Young Shin[2, †], Seong Su Hong[1], Young-Rak Cho[1], Ju-Hyoung Park[2], Dong-Wan Seo[2], Joa Sub Oh[2], Jae-Shin Kang[3], Jae Ho Lee[3] and Eun-Kyung Ahn[1,*]

[1]*Bio-Center, Gyeonggido Business & Science Accelerator (GBSA), Suwon 16229, Republic of Korea.*

[2]*College of Pharmacy, Dankook University, Cheonan 31116, Republic of Korea.*

[3]*Biological Genetic Resources Utilization Division, National Institute of Biological Resources, Incheon 22689, Republic of Korea.*

SUPPORTING INFORMATION

List of Supporting Information **Page**

Figure S1. The ^{1}H NMR spectrum of **1** …..…………

S 3

Figure S2. The ^{13}C NMR spectrum of **1**...….........…......

S 3

Figure S3. ESI-MS spectrum of **1**...…..........…......

S 4

Figure S4. The ^{1}H NMR spectrum of **2** …..…………

S 5

Figure S5. The ^{13}C NMR spectrum of **2**…...…......

S 5

Figure S6. ESI-MS spectrum of **2** …...…………

S 6

Figure S1.

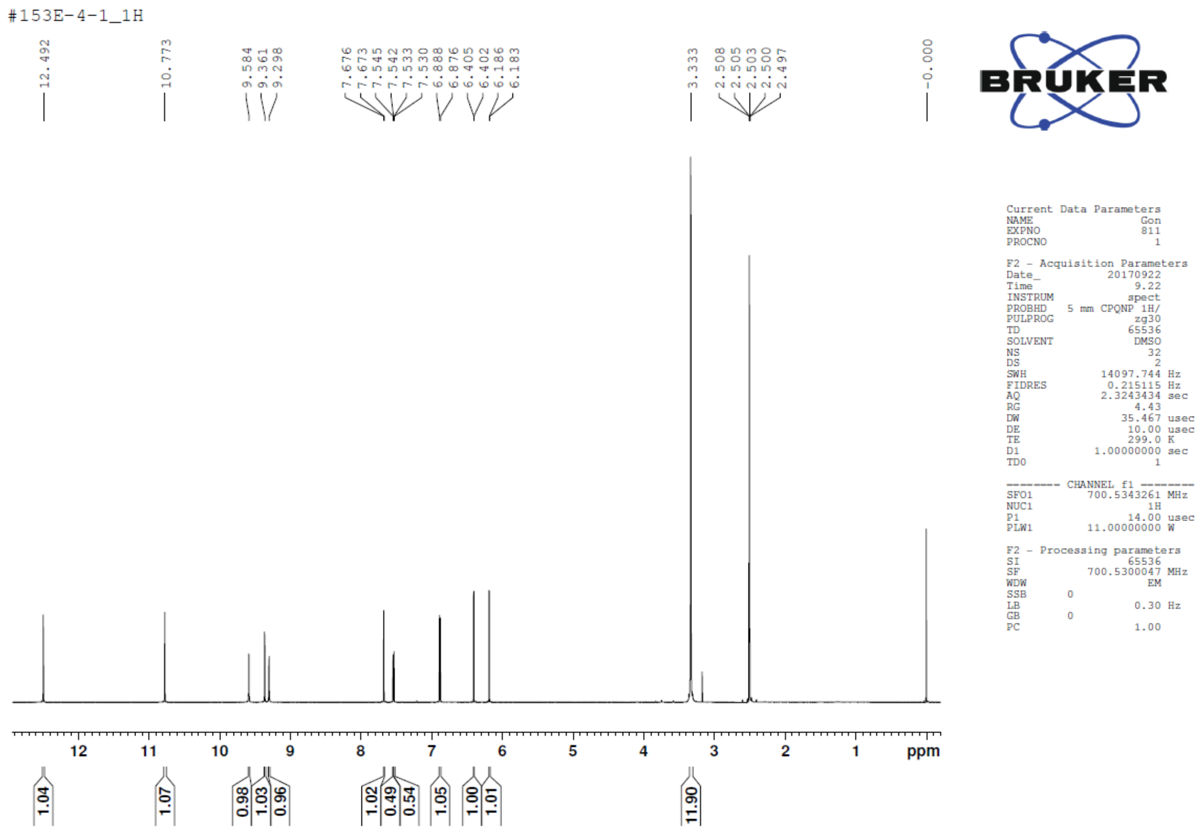

Figure S2.

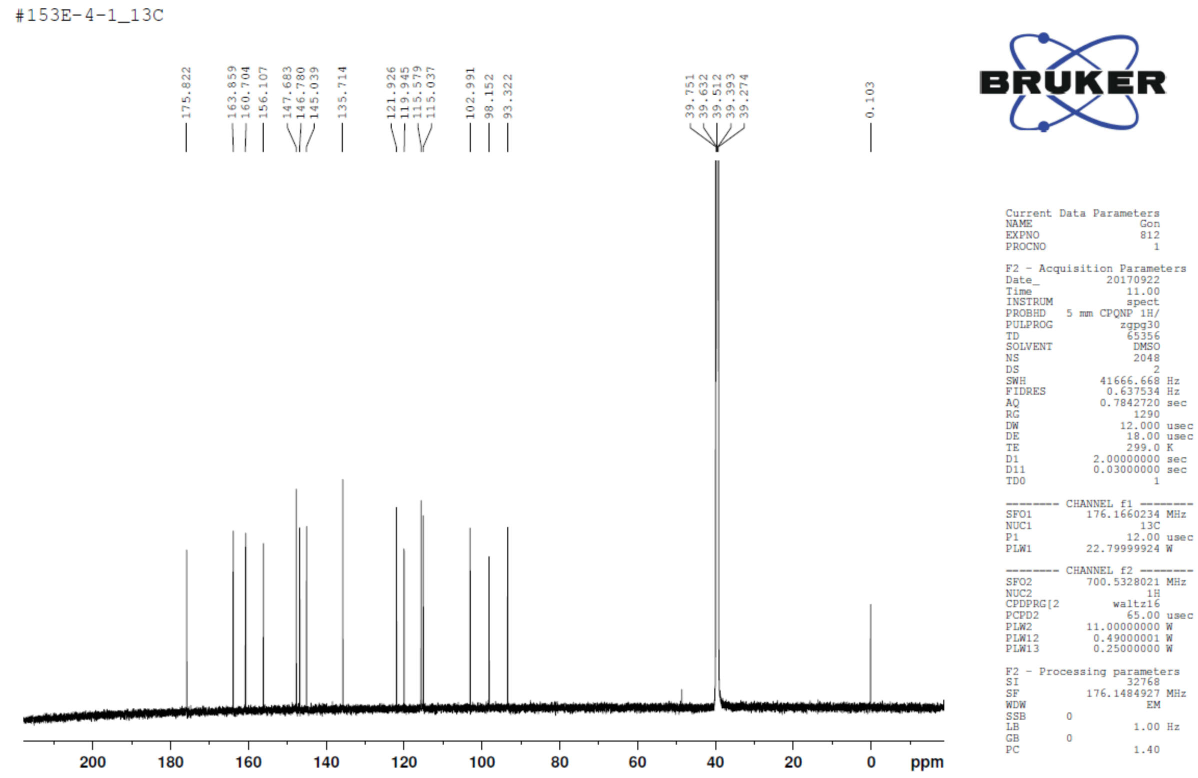

Figure S3.

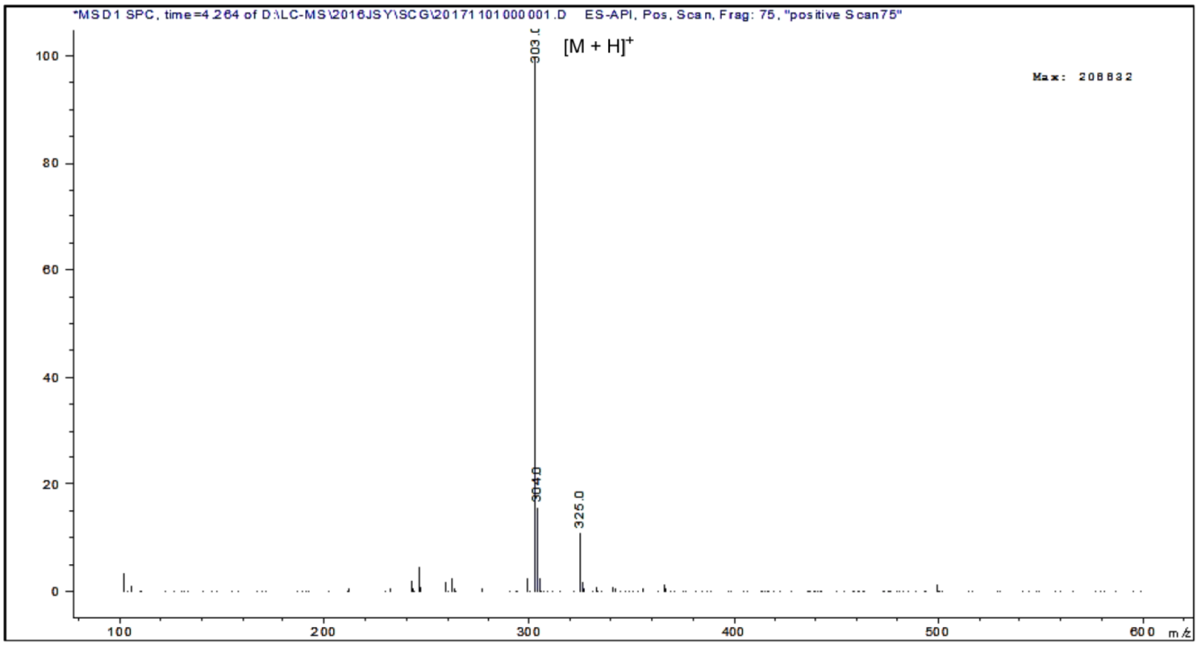
*MSD1 SPC, time=4.264 of D:\LC-MS\2016JSY\SCG\20171101000001.D ES-API, Pos, Scan, Frag: 75, "positive Scan75"
303.0
[M + H]+
Max: 208832
304.0
325.0
100
80
60
40
20
0
100
200
300
400
500
600
m/z

Figure S4.

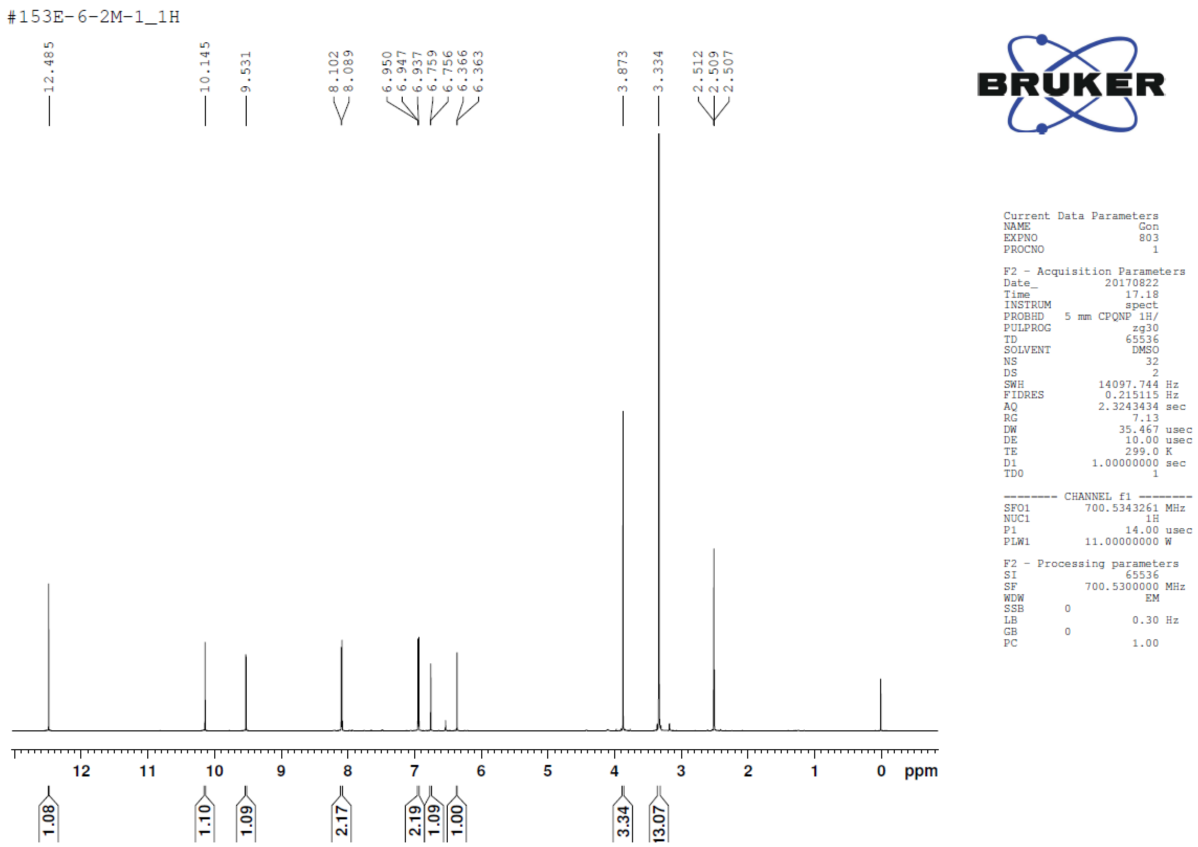

Figure S5.

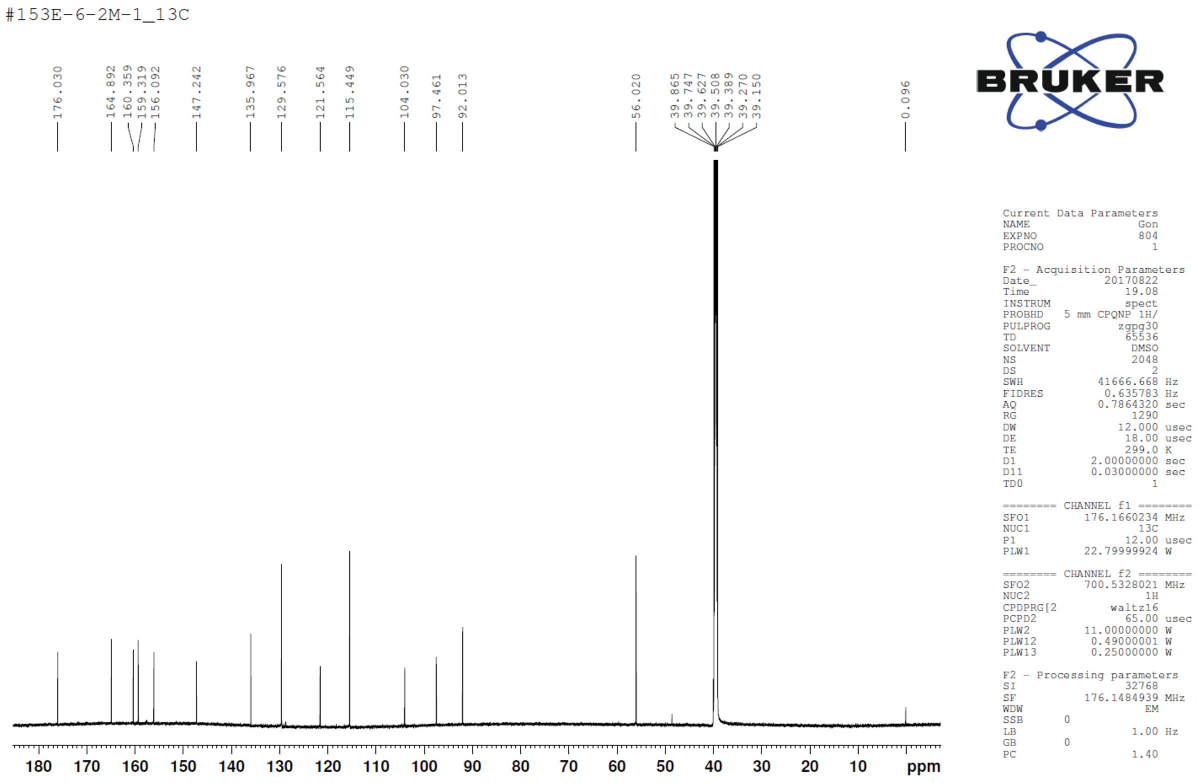

Figure S6.

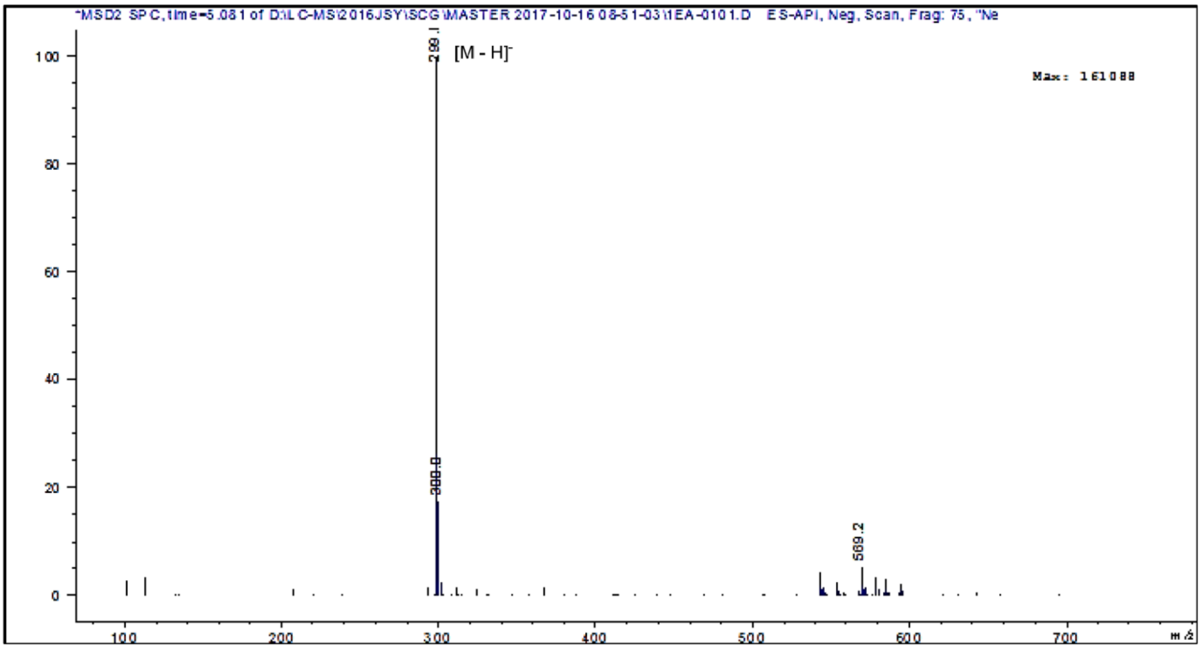
*MSD2 SPC, time=5.081 of D:\LC-MS\2016JSY\SCG\MASTER 2017-10-16 08-51-03\1EA-0101.D ES-API, Neg, Scan, Frag: 75, "Ne
299.1
[M - H]⁻
Max: 161088
569.2
m/z
